# Challenges to Design and Develop of DNA Aptamers for Protein Targets. I. Optimization of Asymmetric PCR for Generation of a Single Stranded DNA Library 

**Published:** 2014

**Authors:** Maryam Tabarzad, Bahram Kazemi, Hossein Vahidi, Reza Aboofazeli, Soraya Shahhosseini, Nastaran Nafissi-Varcheh

**Affiliations:** a *Department of Pharmaceutical Biotechnology, School of Pharmacy, Shahid Beheshti University of Medical Sciences, Tehran, Iran.*; b *Student*᾽*s Research Committee, Shahid Beheshti University of Medical Sciences, Tehran, Iran.*; c *Cellular and Molecular Biology Research Center, Shahid Beheshti University of Medical Sciences, Tehran, Iran. *; d *Biotechnology Department, School of Medicine, Shahid Beheshti University of Medical Sciences, Tehran, Iran.*; e *Department of Pharmaceutics, School of Pharmacy, Shahid Beheshti University of Medical Sciences, Tehran, Iran.*; f *Department of Pharmaceutical Chemistry, School of Pharmacy, Shahid Beheshti University of Medical Sciences, Tehran, Iran . *

**Keywords:** Aptamer, Single stranded DNA, Random pool, SELEX, Asymmetric PCR

## Abstract

Aptamers, or single stranded oligonucleotides, are produced by systematic evolution of ligands by exponential enrichment, abbreviated as SELEX. In the amplification and regeneration step of SELEX technique, dsDNA is conversed to ssDNA. Asymmetric PCR is one of the methods used for the generation of ssDNA. The purpose of this study was to design a random DNA library for selection of aptamers with high affinity for protein targets and develop an efficient asymmetric PCR amplification. Thus, the influence of factors including annealing temperature, number of amplification cycles, primer ratio, Mg^2+^ concentration and the presence of a PCR enhancer on the amplification of the desired product were evaluated. Results obtained by agarose gel electrophoresis showed that the annealing temperature of 64 °C, Mg^2+^ concentration of 0.25 mM, reverse to forward primer ratio of 15:1, amplification cycle of 25 and the presence L-ectoin as a PCR enhancer with the concentration of 0.4 M were the optimal conditions. Our results supported that the yield of this type of ssDNA production is sufficient for combinatorial screening of aptamers.

## Introduction

Aptamers are short DNA or RNA oligonucleotides with high, specific affinity to a special target. The name was originated from *aptus *that means “to fit” and *meros *that shows the polymer identity of oligonucleotides (1, 2). Aptamer characteristics provide prominent potential applications in multiple fields.These nucleic acid ligands are completely generated through *in-vitro *process for a wide range of targets from small molecules and ions to large proteins and cells and even whole organism or tissue. Their chemical modifications could be easily performed to improve the intended specificity. Meanwhile, they keep their stability against various conditional stresses and show lower toxicity and immunogenicity than other specific ligands *e.g*. monoclonal antibodies. Because of the high specificity, adaptability, and ease of modification, aptamers have been used in a broad range of applications, including affinity purification, drug discovery, high-throughput screening, drug delivery, medical diagnostics and biosensors (3-5).

In molecular biology, there are several methods that could help researchers for *in-vitro *evolution of single stranded oligonucleotide pools to high affinity ligands like aptamers. *In-vitro *evolution is the experimental process in which large random-sequence pools of RNA or DNA are used as the starting point and particular nucleic acid sequences with higher affinity to an intended target are identified as aptamers. This type of selection and evolution is termed SELEX (*systematic evolution of ligands by exponential enrichment*) (6-9).

Random pools of single stranded oligonucleotides are the critical agents in the SELEX process and therefore fundamental points should be considered to design an effective one (2). The initial random pool of oligonucleotides is always DNA which is synthesized chemically or derived from genome sequences. Amplification of these DNA oligonucleotides is also an important part of their handling. For DNA libraries, single stranded DNA (ssDNAs) are produced at each round of SELEX and hence the most critical step of amplification is the conversion of the double stranded DNA (dsDNA) to ssDNA, since only ssDNAs can fold into varient 3D structures that are necessary for target binding, whereas dsDNAs have only the double helix configuration. There are several methods available in generating ssDNA from the corresponding dsDNA (10) including asymmetric PCR (11), biotin–streptavidin separation (12), lambda exonuclease digestion (13) and size separation on denaturing-urea polyacryl amide gel electrophoresis (14). Nevertheless, high costs and incomplete enzymatic processes are the major limitations of the digestion methods, electrophoresis is time-consuming and biotin-streptavidin separation is also very expensive (15).

Each amplification step with Taq DNA polymerase in SELEX process could raise the diversity of random library and subsequently improves the aptamer selection (16, 17). It is expected that asymmetric PCR to be a good method. Lower cost and ease of technique highly support this choice. Asymmetric PCR is also beneficial for intermittent amplification of very minute amounts of DNA after each round of SELEX process and before starting the next rounds (10). 

Homogeneity of length in random ssDNA pool is one of the standard goals of amplification process. Shorter or longer sequences which are defined as non-specific products result from the presence of secondary structures that disturb polymerization reaction or from product-product or primer-product annealing, respectively (18, 19). The application of non-equal amounts of the reverse and forward primers for amplification in asymmetric PCR method (18) could provide a diverse SELEX pool of uniform ssDNA structures. Several reports are available on the attempts made to reduce the formation of non-specific products during ssDNA amplification by asymmetric PCR (20).

The factors that influence the specificity and amount of amplified DNA by PCR are annealing temperature, MgCl2 concentration and the number of amplification cycles. For ssDNA production by asymmetric PCR, the primers concentration ratio is also important (21, 22). Asymmetric PCR routinely runs after a symmetric PCR for ssDNA amplification during aptamer selection by SELEX. Considering the importance of ssDNA production and amplification and its direct effects on final selected aptamer characteristics, the optimization of asymmetric PCR for each individual library is critical. In the present study, we planned to design random DNA library for further aptamer selection and amplify the library by asymmetric PCR. In this regard, the influence of factors including annealing temperature, amplification cycles numbers, primer ratio, Mg^2+^ concentration and the presence of a solute PCR enhancer on quantity and quality of desired product were evaluated.

## Experimentals

*Materials*

PCR set including Taq DNA polymerase (5IU/μL), PCR 10x buffer (500 mM KCl and 200mM Tris-HCl, pH 8.4) and MgCl2 solution (100 mM) were purchased from SinaClone (Iran). HPLC grade random single stranded DNA library and forward and reverse primers were synthesized by Metabion Company (Germany) at the concentration of 1 μM. TAE 10x buffer (1x buffer contains 40 mM Tris, 20 mM acetic acid and 1 mM EDTA) was obtained from Fermentas Company (Canada). L-Ectoin was purchased from Biomol (Germany). 50-1500bp DNA ladder and 6x loading dye (bromophenol blue and xylene cyanol) were prepared from GeneOne Company (Germany) and Vivantis Company (USA), respectively. SafeStain dye (a kind of Sybrgreen dye) and molecular grade agarose were obtained from SinaClone (Iran). Purified water with a Millipore system (USA) was applied for polymerization reactions.

*Asymmetric PCR*

In this study, the DNA template for amplification process by asymmetric PCR was a random oligonucleotide pool with a sequence of 5’-GGTGTTACTCTTCATGTGGATCCG(N30)AGAATTCAGCACCCTAGCCTCGT-3’ in which N represents 30 nucleotides of random sequence region with equal proportions of A, C, G and T. Forward and reverse primers were 5’-GGTGTTACTCTTCATGTGGATCCG-3’ and 5’-ACGAGGCTAGGGTGCTGAATTCT-3’, respectively. The library and primers were designed by GeneRuner^®^ software. 5 ng of random DNA pool was used as starting template. The thermocycling program comprised four basic steps, including the initial DNA denaturation step at 95 °C for 3 min, different cycles of DNA denaturation at 95 °C for 30 sec, primer annealing at various temperatures for 45 sec and DNA polymerization at 72 °C for 30 sec. Final amplification was performed at 72 °C for 5 min in order to complete the polymerization process. Primers and deoxynucleotide triphosphate (dNTPs) were added at 40pmol per reaction and 0.2 mM concentration, respectively. The volume of the PCR reaction was 30 μL. Gradient PCR was run at annealing temperature range of 60-70 °C. For asymmetric PCR reactions, different ratios of reverse to forward primer from 5:1 to 20:1 were evaluated whereas the dominant primer amount per reaction was kept constant at 40pmol. A Flex thermal cycler from Analytik Jena Company (Germany) was applied for all PCR experiments.

*Gel electrophoresis and analysis*

Products of amplification were loaded on 3% agarose gel. SafeStain dye was added to agarose at pre-casting step with a volume ratio of 1:10,000. Electrophoresis was run at 70 V for 30 min at room temperature, using a horizontal multiSUB tank equipped by a power supply (Cleaver Company, UK). 10 μL of amplification product plus 2 μL of loading dye buffer were loaded on agarose gel. Image of gels was captured with G:Box^®^ and GeneSnap software (SynGene company, UK) at 254 nm. The intensities of bands were compared using GeneTool^®^ (SynGene Company, UK).

## Results and Dscussion

SELEX process includes iterative rounds of selection and amplification. In the process of DNA aptamer selection, production of ssDNA is a critical step of library amplification. Among several options, asymmetric PCR is the most economic method for the production of ssDNA from very low amount of starting template between SELEX rounds. Access to the higher levels of desired ssDNA product following an asymmetric PCR run makes it possible to enter the amplification products into the next rounds directly and without any purification step, except phenol-chloroform extraction and ethanol precipitation. It should be noted that although the optimization of the amplification process is unique for each individual random oligonucleotide pool and it is not possible to use the same conditions for all pools, however, there are some common points that could help optimize the required conditions by a rational design, if considered. Therefore, optimization of the amplification conditions would be first step in combinatorial screening of a random DNA library for aptamer selection. Several investigations have been reported in the literature, regarding the improvement of ssDNA amplification efficacy. One research group has applied single-primer-limited amplification method to generate ssDNA from a random DNA pool in which the primers were added to polymerization reaction in separate steps (20). PCR enhancers (for example dimethyl sulfoxide and betaine) have also been also suggested to optimize the amplification process (19).

In this study, a random DNA oligonucleotide pool with a sequence of 74 nucleotides in length was designed for the selection of aptamers with high affinity for protein targets and the influence of various factors, including the annealing temperature, magnesium chloride concentration, primer type and ratio, amplification cycles and enhancer was evaluated in an attempt to optimize the asymmetric PCR.

*Effect of annealing temperature*

In the amplification cycles of PCR process, annealing temperature has direct effect on the correct primer annealing and thus the polymerization of correct products. During random DNA pool polymerization in aptamer selection process, there are chances of primer annealing to the random regions due to the possible sequence similarities. Although some investigations have shown that the annealing temperature did not have significant effect on the yield of product in the amplification step (10), however, to minimize the possibility of primer annealing to other roughly similar sequences in random regions, selecting a proper temperature for the polymerization step seems to be necessary.

**Figure 1:**
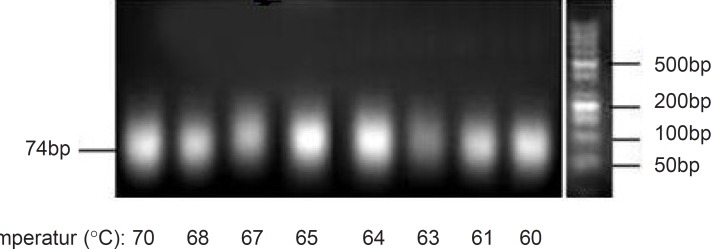
Effect of various annealing temperatures on the PCR products. Results were obtained from 3% agarose gel electrophoresis (position of dsDNA74bp is marked). Annealing temperatures were applied in a gradient manner for ssDNA pool amplification by symmetric PCR.

For this purpose in this study, the effect of annealing temperature on PCR products was analyzed. Considering melting temperature (Tm) of the used forward and reverse primers (65 °C for both), a series of amplification process were run at different annealing temperatures ranging from 60 °C to 70 °C in a gradient PCR manner. Magnesium chloride concentration and the primer amount were kept constant at 0.25 mM and 40pmol, respectively. Analyses of amplification products after 20 cycles were performed and corresponding results were shown in Figure 1. Similar to the previous report (10), no obvious differences in the intensities of desired product bands were observed and therefore the annealing temperature was not considered to be a critical factor in the amplification process. Nevertheless, the incorrect primer annealing at lower temperatures may result in an increase of byproducts, and also lower primer annealing at high temperatures decreases the product yield (21). Therefore, based on the results obtained, the annealing temperature of 64 °C was selected for our pool.

*Effect of magnesium chloride concentration*

**Figure 2:**
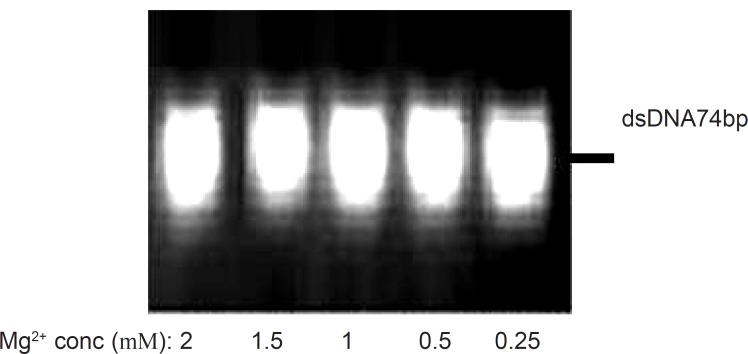
Effect of various magnesium chloride concentrations on the PCR products. Results were obtained from 3% agarose gel electrophoresis (position of dsDNA74bp is marked). symmetric PCR amplification of ssDNA pool was run after 20 cycles, at the annealing temperature of 64 °C

The role of magnesium ion on the activity of polymerases has been well understood (23) and for this reason, the optimization of Mg^2+^ concentration is suggested for successful amplification. In this study, different concentrations of magnesium chloride (0.25 mM to 2 mM) were analyzed by five amplification reactions with 40pmol of forward and reverse primers and 20 cycles of amplification at the annealing temperature of 64 °C. Figure 2 depicts the effect of various magnesium chloride concentrations on the PCR products after 20 cycles, obtained from 3% agarose gel electrophoresis. Although Mg^2+^ cations could stabilize the undesired secondary structures of DNA sequences and accordingly result in the production of some byproducts with smaller lengths (19), however, it was observed in our study that the yield of desired product was not influenced by MgCl2 in the concentration range of 0.25 mM to 2 mM.

*Effect of primer type and ratio*

It has been reported that using the forward or reverse primer as the dominant primer during amplification process would have significant influences on the PRC products (20). In this study, the reverse or forward primers were initially added into two separate asymmetric PCR tubes at the primer ratio of 5:1. The amplification was then run for 20 cycles at the annealing temperature of 64 °C and in the presence of 0.25 mM MgCl2.

**Figure 3:**
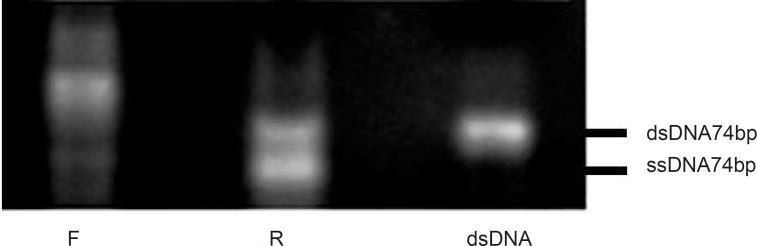
Effect of various primers on the asymmetric PCR products. Results were obtained from 3% agarose gel electrophoresis (positions of ssDNA74bp and dsDNA74bp are marked). Amplification of ssDNA pool at the F:R primer ratio of 5:1 (lane F), at the R:F primer ratio of 5:1 (lane R) and at the F:R primer ratio of 1:1 (lane dsDNA).

**Figure 4:**
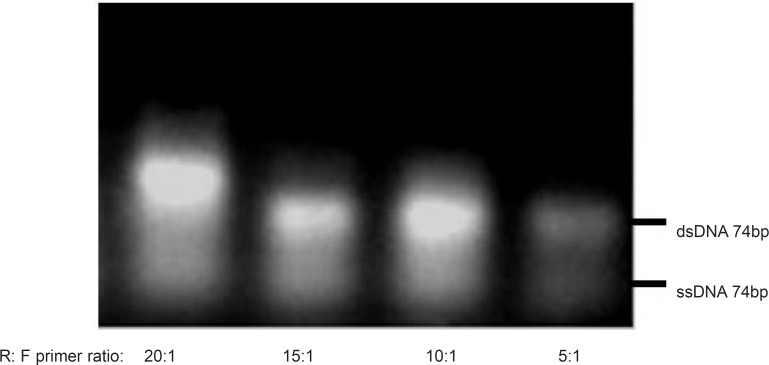
Effect of various R: F primer ratios on the asymmetric PCR products. Results obtained from 3% agarose gel electrophoresis (positions of ssDNA74bp and dsDNA74bp are marked). Asymmetric PCR amplification of ssDNA pool was performed at annealing temperature of 64 °C and 0.25 mM magnesium chloride

As shown in Figure 3, the dominancy of the forward primer (F) led to the production of non-specific sequences, but when the reverse primer (R) was considered as dominant, the amplification and ssDNA production seemed to be desirable. Thus, for the following experiments, reverse primer was selected as the dominant at the concentration of 40pmol per reaction and the effect of different ratios of R: F ranging from 5:1 to 20:1 were evaluated (Figure 4). The optimum ratio was found at 15:1. At ratios lower than 15:1, the ssDNA yield was lower and the level of double stranded sequences was higher in comparison with the single stranded products. Although the enhancement of dominant primer was accompanied by higher concentration of the desired products, at the R: F ratio of 20:1, new bands of the higher length byproducts also appeared which may be attributed to product-product or product-primer annealing. Based on the results obtained, the ratio of 15:1 was selected for further experiments in this study.

*Effect of amplification cycles*

It has been reported that increasing the number of amplification cycles of PCR results in more product formation and in contrast, at very high number of cycles, primers are exhausted and the annealing of product and template in undesired manner are performed (18). In other words, an optimum upper limit is required for obtaining desired yield.

**Figure 5:**
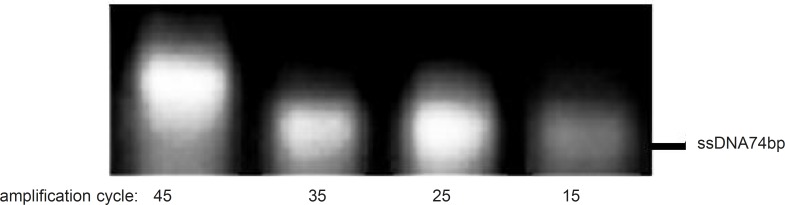
Effect of different amplification cycles on the asymmetric PCR products. Results obtained from 3% agarose gel electrophoresis (position of ssDNA74bp is marked). Asymmetric PCR amplification of ssDNA pool was performed at R:F primer raio of 15:1, annealing temperature of 64 °C and 0.25mM magnesium chloride

To examine the effect of this factor, asymmetric PCR reactions were run at different cycles of amplification (15 to 45 cycles by intervals of 10 cycles), whereas the R:F primer ratio, annealing temperature and Mg^2+^ concentration were kept constant at 15:1, 64 °C and 0.25 mM, respectively. After 25 cycles of amplification, non-specific products were detectable in the gel (Figure 5). Thus, 25 cycles of amplification was considered as the optimum number of cycles.

**Figure 6:**
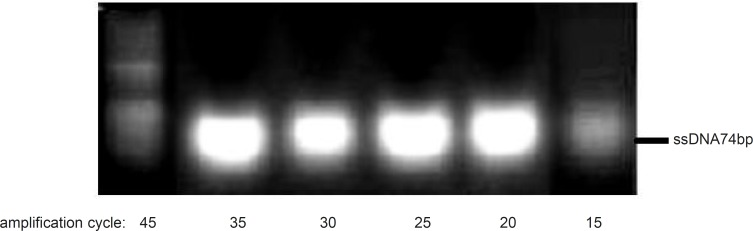
Analyses of asymmetric PCR products, obtained by the use of only reverse primer at different amplification cycles. Results obtained from 3% agarose gel electrophoresis (position of ssDNA74bp is marked).

Although it is expected that increasing the amplification cycle would result in higher yield, however, an increase of the product level and a decrease of the primers amounts during the process (especially in random pools) could enhance the risk of product-product annealing or product-template annealing which subsequently leads to the formation of nonspecific products (18). In this study, effect of polymerization cycles on the amplification of the desired product by using only 40pmol of reverse primer was analyzed. Our result supported that the amplification in the presence of one primer up to 35 cycles improved the formation of the desired product (Figure 6).

*Effect of enhancer*

There are a few reports on the effects of compatible solutes (*i.e*. small organic zwitterionic substances) on dsDNA melting and amplification enhancement of GC-rich templates. Furthermore, solutes may influence the polymerase stability during the PCR steps. In random oligonucleotide pool, presence of some GC-rich sequences is also possible and enhancers might improve their amplification (19, 24-26).

Theoretically, compatible solutes rather than being enzyme activity stabilizer, could act as PCR enhancers and change DNA secondary structure and melting temperature especially in GC-rich sequences, through decreasing the ionic interactions. Accordingly, they diminish the effect of cations (*e.g*. Mg^2+^ and Na^+^) on DNA melting temperatures (26). In SELEX process, binding buffers which are applied for the incubation of DNA library and target molecules usually have high ionic concentrations and if these buffers are transferred to the PCR reaction media, they could influence the amplification efficacy. Meanwhile, the stabilization of the secondary structures of DNA by available cations disturbs the process. Therefore, solutes might help in increasing the full length sequence amplification by opening the stem-loop structures in ssDNAs (19, 26).

**Figure 7:**
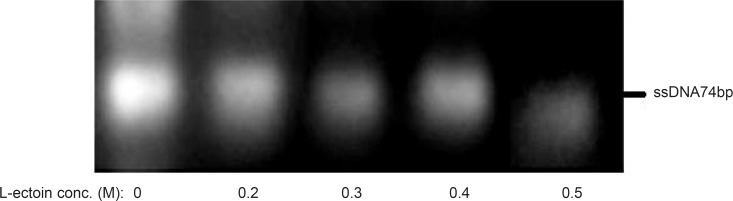
Effect of various concentrations of L-ectoin as an enhancer on the asymmetric PCR products. Results obtained from 3% agarose gel electrophoresis (position of ssDNA74bp is marked). Asymmetric PCR amplification of ssDNA pool was performed at R:F primer raio of 15:1, annealing temperature of 64 °C and 0.25 mM magnesium chloride for 25 cycles

Effect of compatible solutes as PCR enhancers on the amplification process might be due to effect of these compounds on melting temperatures of dsDNA especially in GC-rich sequences. In random oligonucleotide pools, the GC contents of different sequences oligonucleotides are not homogenous and hence the amplification efficacy of each individual sequence could be different (19). The change in amplification yield of various sequences magnifies some sequences more than the others and offers biases in random screening. Adding the solutes to polymerization reactions media of ssDNA could open the secondary structures and an increase of desired full length products intensifies the homogenous amplification of all sequences. The positive effects of the combination of DSMO and betaine on the formation of full length products by PCR in random oligonucleotides pool amplification have been reported (19). L-ectoin is also a compatible solute that its effect on PCR reaction has been previously evaluated. Schnoor *et al. *showed that L-ectoin could have PCR enhancing effect at the concentration of 0.25-0.5 M, which is much lower than that required for betaine to produce the same enhancing effect (*i.e*. 2M) (26). To evaluate the solute effect on decreasing the amount of nonspecific products, six concentrations of L-ectoin (0.2 – 0.5 M) were added to the asymmetric PCR medium and the polymerization reactions were executed at R:F primer ratio of 15:1, 25 amplification cycles, annealing temperature of 64 °C and 0.25 mM MgCl2. (Figure 7). In contrast to the previous claim (26) that L-ectoin would increase the byproduct of amplification, at our studied concentrations, no increase in byproducts formation was observed.

The results of asymmetric PCR in presence of L-ectoin as a compatible solute for the enhancement of amplification indicated a concentration- dependent effect. At concentrations more than 0.4M, the total product formation decreased whereas at lower concentrations, non-specific bands were observed.

In general, single stranded DNA production and amplification are highly effective steps in aptamer selection by SELEX process. *In-vitro *screening of aptamers is performed by several rounds; each includes target binding and partitioning followed by sequential selection and amplification. In general, this is a time consuming process. Thus, using simple and non-complicated methods in different steps of selection and amplification is a crucial consideration for minimizing the cost and time of the whole process. If in one step of the asymmetric polymerization, a good yield of ssDNA was obtained, it might be possible to eliminate PCR step prior to asymmetric PCR during SELEX process. In this study, using one step asymmetric PCR for ssDNA amplification was evaluated. According to the results obtained, the yield of this type of ssDNA production is sufficient for combinatorial screening of aptamers. 

*Conclusion*

Aptamers have attracted much attention due to their capability of binding to target molecules with high affinity and specificity. These macromolecules have been extensively studied in the literature, concerning their application in the development of new drugs and drug delivery systems. Isolation of aptamers with high affinity for a specific target is executed by SELEX. The most important and critical step in this engineering process is the conversion of dsDNA to ssDNA, through which DNA aptamers are generated. Among the various techniques developed for this process, asymmetric PCR has found more application. In this research, we decided to optimize the asymmetric PCR for the generation of an ssDNA library. Therefore, access to an efficient approach, considering the cost and ease of procedure, could be promising for further improvement of the success rate in DNA aptamer selection for protein targets.
